# Supplementary material for: Broad H3K4me3 Domain Is Associated with Spatial Coherence during Mammalian Embryonic Development
Source: bioRxiv. 2023 Dec 12:2023.12.11.570452. Preprint. [Version 1] doi: 10.1101/2023.12.11.570452 (PMC10760050; doi:10.1101/2023.12.11.570452)
Supplement: 1 [file NIHPP2023.12.11.570452V1-supplement-1.pdf]

bioRxiv preprint doi: https://doi.org/10.1101/2023.12.11.570452; this version posted December 12, 2023. The copyright holder for this preprint (which was not certified by peer review) is the author/funder, who has granted bioRxiv a license to display the preprint in perpetuity. It is made available under aCC-BY 4.0 International license.

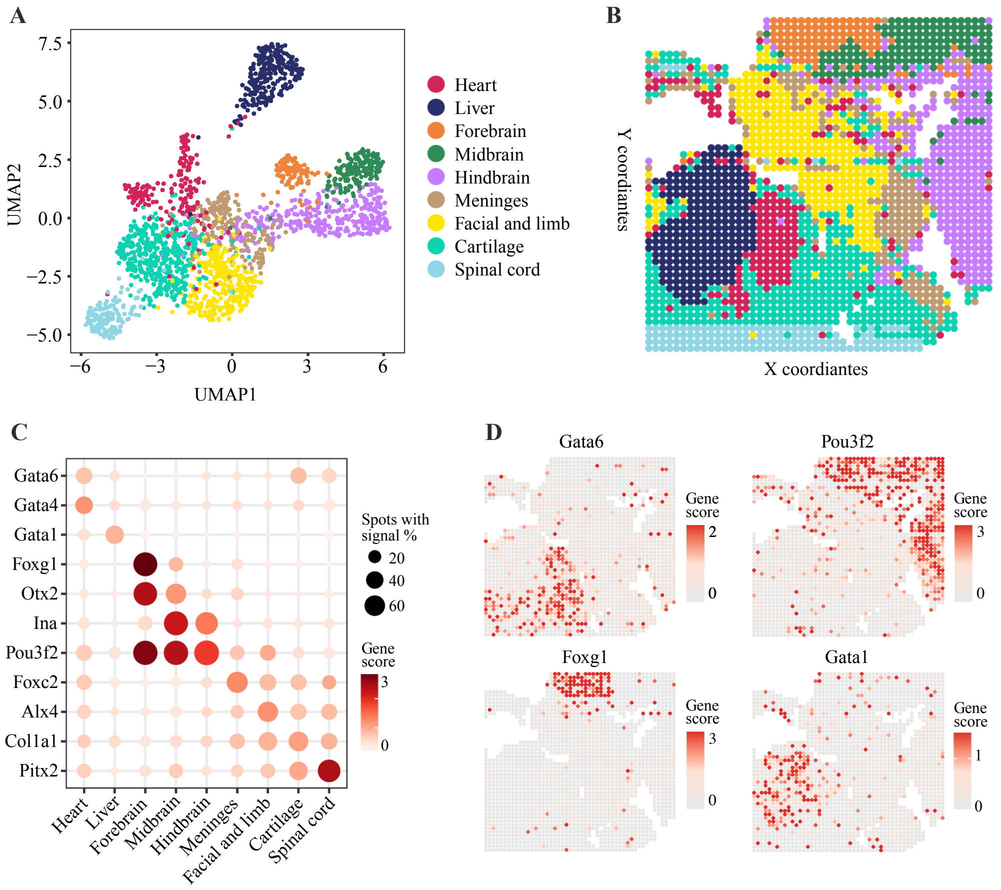

Figure S1. Clusters annotation based on genome-wide H3K4me3 profiles. Related to Figure 1.

(**A-B**) UMAP and spatial distribution of clusters identified by clustering analysis. Each cluster is represented by a different color.

(**C**) Clusters specific H3K4me3 signals for representative marker genes.

(**D**) Spatial patterns associated with representative genes.

bioRxiv preprint doi: https://doi.org/10.1101/2023.12.11.570452; this version posted December 12, 2023. The copyright holder for this preprint (which was not certified by peer review) is the author/funder, who has granted bioRxiv a license to display the preprint in perpetuity. It is made available under aCC-BY 4.0 International license.

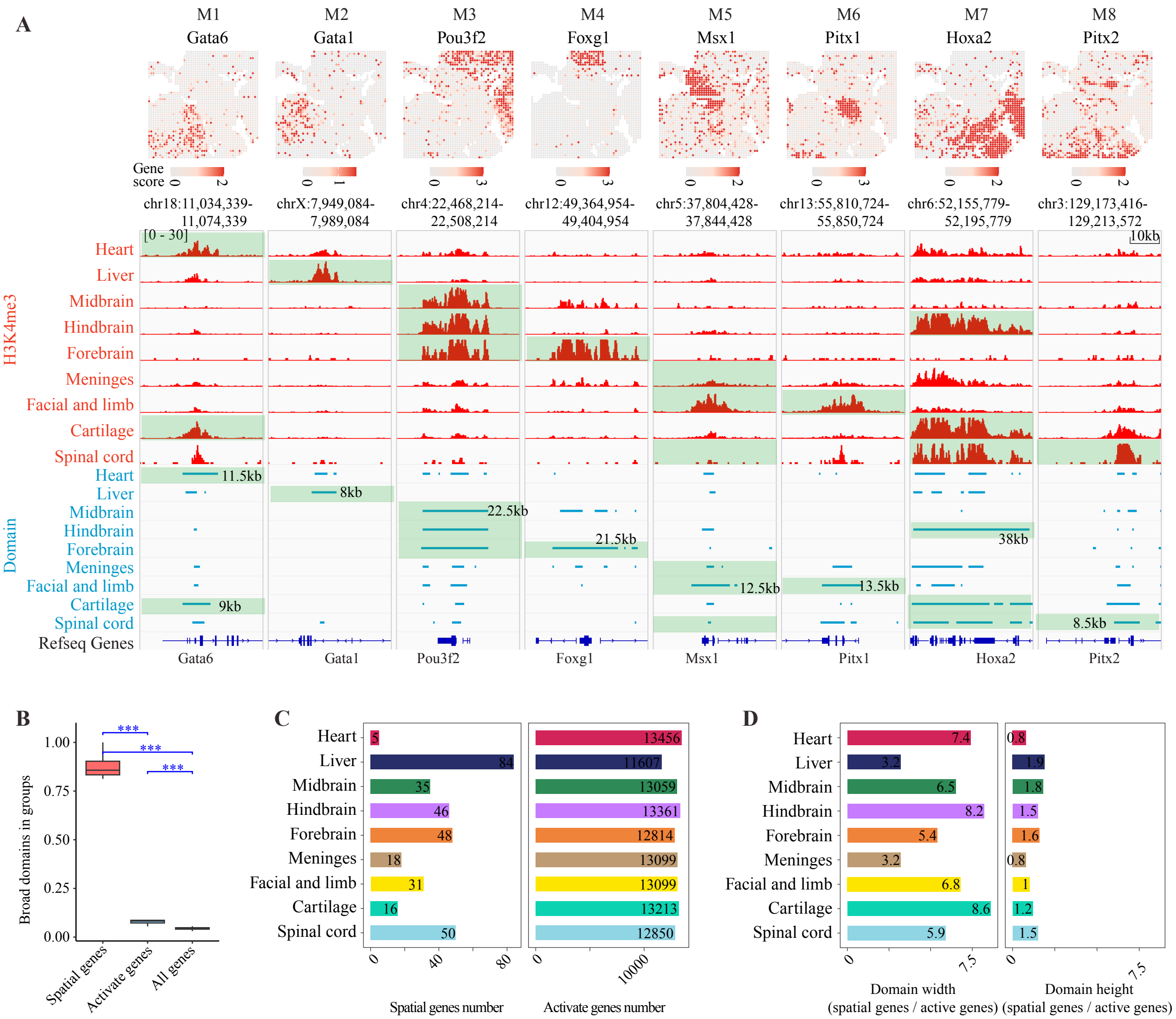

Figure S2. Broad H3K4me3 domains are generally associated with spatial genes. Related to Figure 2.

(**A**) Spatial distribution, genome browser density, and domain width of spatial genes in modules. Colors on heatmap represent the H3K4me3 gene scores. Green shadows represent the modules related clusters.

(**B**) Comparisons of the H3K4me3 domain enrichment between spatial genes, activate genes, and all genes (genome-wide). Y axis is the number of broad domains divided by the number of genes in each cluster.

(**C**) Barplots showing the number of spatial genes and activate genes within each cluster.

(**D**) Barplots showing the ratio of domain width and height between spatial genes and activate genes.

bioRxiv preprint doi: https://doi.org/10.1101/2023.12.11.570452; this version posted December 12, 2023. The copyright holder for this preprint (which was not certified by peer review) is the author/funder, who has granted bioRxiv a license to display the preprint in perpetuity. It is made available under aCC-BY 4.0 International license.

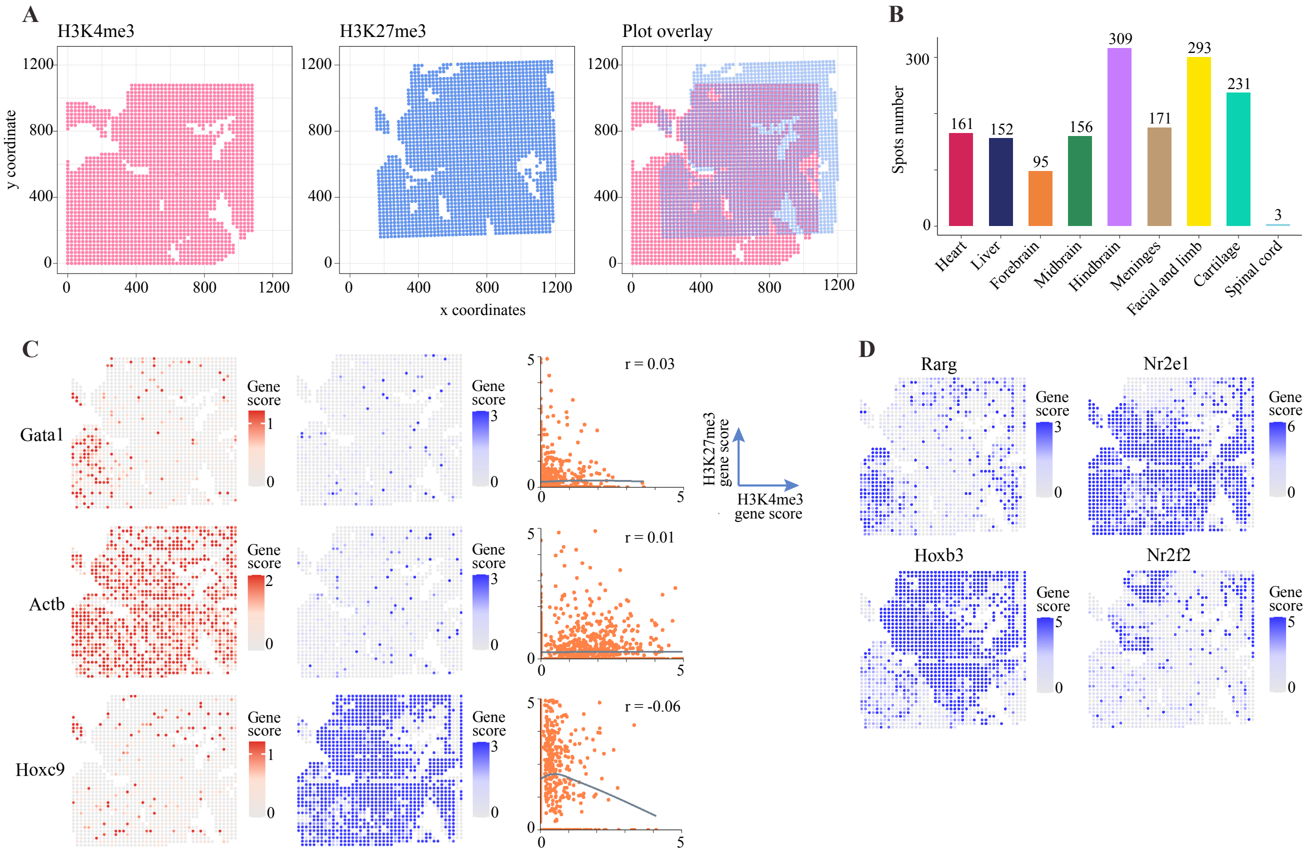

Figure S3. Alignment between H3K4me3 and H3K27me3, and selected genes of H3K27me3. Related to Figure 3.

(**A**) Overlay of the aligned H3K27me3 to H3K4me3 signals.

(**B**) The number of spots kept after alignment for each cluster as in Figure 1B.

(**C**) Dot plot showing the H3K27me3 signals for representative marker genes as in Figure 1C.

(**D**) Spatial distribution heatmaps of H3K27me3 signals of representative spatial genes.

bioRxiv preprint doi: https://doi.org/10.1101/2023.12.11.570452; this version posted December 12, 2023. The copyright holder for this preprint (which was not certified by peer review) is the author/funder, who has granted bioRxiv a license to display the preprint in perpetuity. It is made available under aCC-BY 4.0 International license.

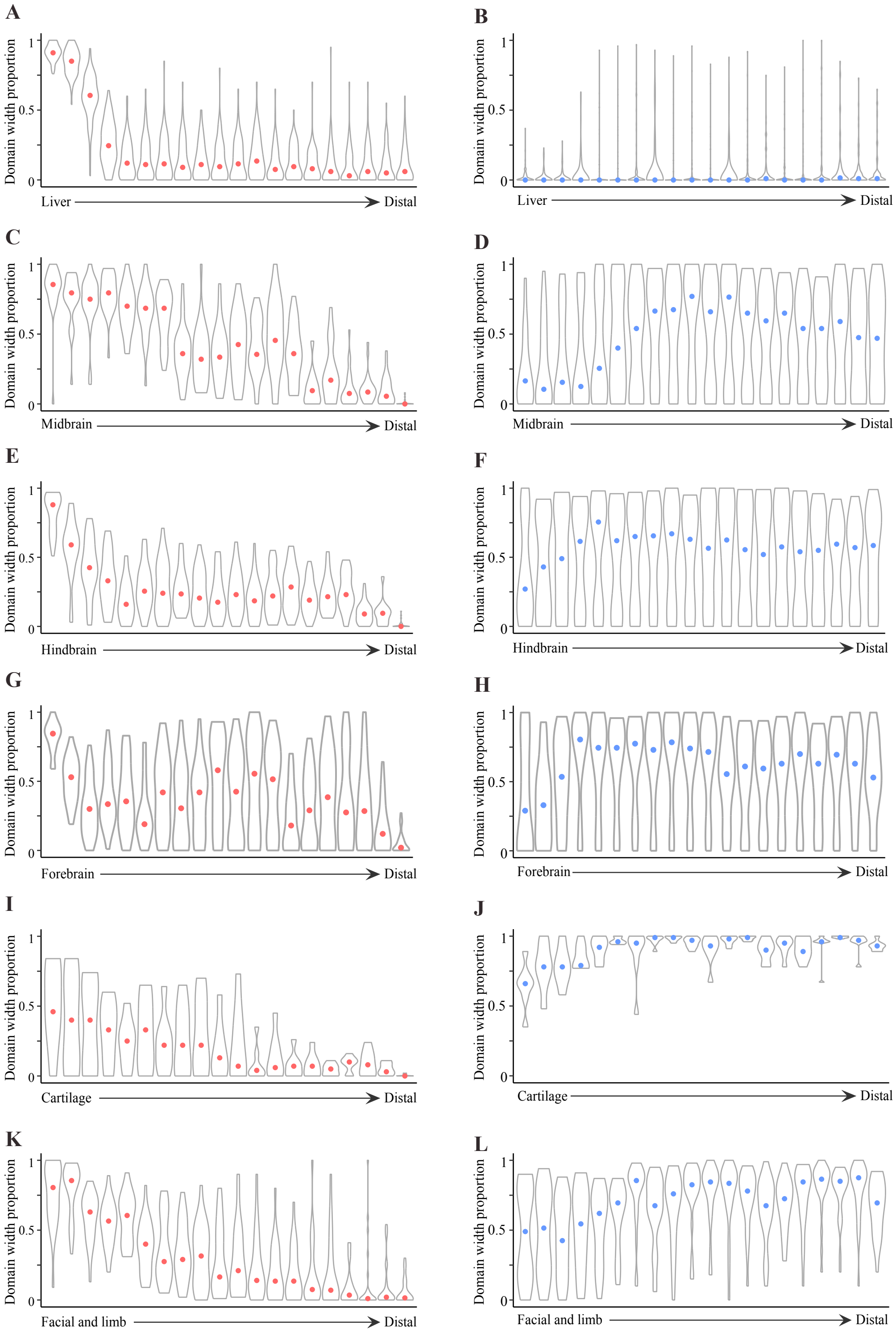

Figure S4. Spatial transition of chromatin states across tissues. Related to Figure 4.
(**A-B**) The distribution of H3K4me3 and H3K27me3 (B) domain width for all spatial genes in the M2 H3K4me3 module across liver.
(**C-D**) Similar to (A-B) but for M3 H3K4me3 module across midbrain.
(**E-F**) Similar to (A-B) but for M3 and M7 H3K4me3 modules across hindbrain.
(**G-H**) Similar to (A-B) but for M3 and M4 H3K4me3 modules across forebrain.
(**I-J**) Similar to (A-B) but for M1 and M7 H3K4me3 modules across cartilage.
(**K-L**) Similar to (A-B) but for M5 and M6 H3K4me3 modules across facial and limb.
